# Efficacy and safety in the use of intraperitoneal hyperthermia chemotherapy and peritoneal cytoreductive surgery for pseudomyxoma peritonei from appendiceal neoplasm: A systematic review

**DOI:** 10.1016/j.clinsp.2022.100039

**Published:** 2022-05-14

**Authors:** Idevaldo Floriano, Antônio Silvinato, João C. Reis, Claudia Cafalli, Wanderley Marques Bernardo

**Affiliations:** a Evidence Based Medicine Center, UNIMED Cooperative, Baixa Mogiana regional, Mogi-Guaçu, SP, Brazil; b Evidence Based Medicine Center, UNIMED Fesp, São Paulo, SP, Brazil; c Guidelines Program of the Brazilian Medical Association, São Paulo, SP, Brazil; d Evidence Based Medicine Center, UNIMED Fesp, São Paulo, SP, Brazil

**Keywords:** Pseudomyxoma peritonei, Intra-abdominal hypertermic chemotherapy, Cecal appendix, Appendiceal, Cytoreductive surgery, HIPEC, CRS, Abdominal carcinomatosis

## Abstract

The objective of this systematic review is to provide efficacy and safety data in the application of Intra-Abdominal Hyperthermia Chemotherapy (HIPEC) and Cytoreductive Surgery (CRS) in patients with Peritoneal Pseudomyxoma (PMP) of origin in the cecal appendix. The databases Medline and Central Cochrane were consulted. Patients with PMP of origin in the cecal appendix, classified as low grade, high or indeterminate, submitted to HIPEC and CRS. The results were meta-analyzed using the Comprehensive Metanalysis software. Twenty-six studies were selected to support this review. For low-grade PMP outcome, 60-month risk of mortality, Disease-Free Survival (DFS), and adverse events was 28.8% (95% CI 25.9 to 32), 43% (95% CI 36.4 and 49.8), and 46.7% (95% CI 40.7 to 52.8); for high-grade PMP, 60-month risk of mortality, Disease-Free Survival (DFS) and adverse events was 55.9% (95% CI 51.9 to 59.6), 20.1% (95% CI 15.5 to 25.7) and 30% (95% CI 25.2 to 35.3); PMP indeterminate degree, 60-month risk of mortality, Disease-Free Survival (DFS) and adverse events was 32.6% (95% CI 30.5 to 34.7), 61.8% (95% CI 58.8 to 64.7) and 32.9% (95% CI 30.5 to 35.4). The authors conclude that the HIPEC technique and cytoreductive surgery can be applied to selected cases of patients with PMP of peritoneal origin with satisfactory results.

## Highlights

• Hyperthermia chemotherapy and cytoreductive surgery in patients with peritoneal pseudomyxoma.

## Introduction

Peritoneal Pseudomyxoma (PMP) was first described by Rokitansky in 1842;^1^ Werth, in 1884,^2^ introduced the term peritoneal pseudomyxoma, describing ovarian mucinous carcinoma and presence of gelatinous ascites "("jelly belly""). In 1901, Frankel described the first case of peritoneal pseuxomyxomatous syndrome resulting from cystic rupture in cecal appendix.

This disease is a rare type of cancer that involves the peritoneal surface, whose most common origin is the cecal appendix, but also occurs in other places such as stomach, colon, meso or ovarian. It is characterized by the large production of mucin, with consequent mucinous ascites.

**Fig. 1:**
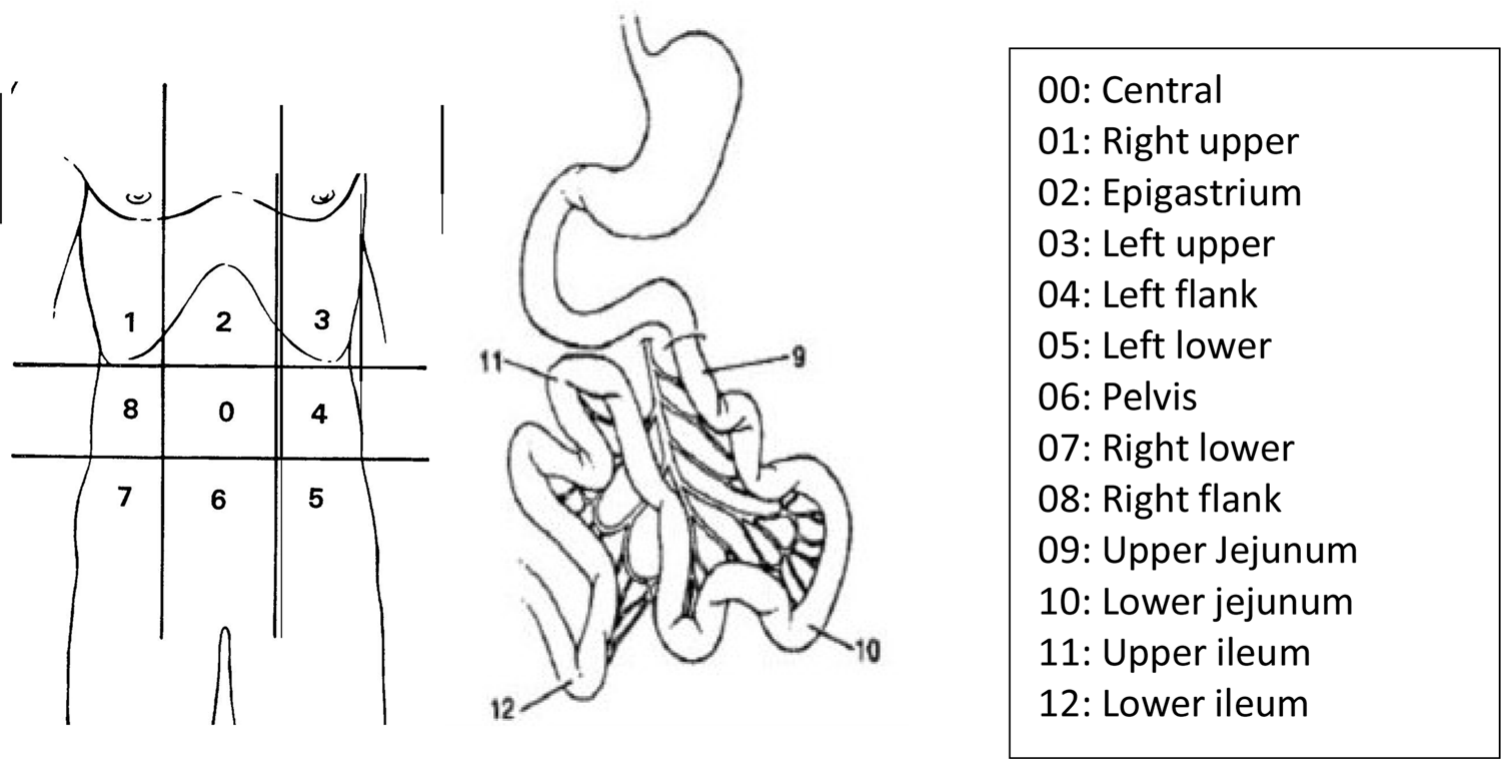
Sugarbaker, Classification of peritoneal carcinomatosis index.^3^ Source: Adapted from Brucher et al.^4^ (p. 2012).

In 1995, Sugarbaker^3^ quantified the dispersion of abdominal disease through numerical values correlated to quadrants of the abdomen, determining the Peritoneal Carcinomatosis Index (PCI), according to the classification below (Fig. 1).

The surgical treatment applied PMP is performed through Peritoneal Cytoreductive surgery (CCP) that can be surgically classified^5^ in:

• CC-0 - No residual tumor (= R0 resection) (en bloc resection);
• CC-1 ‒ < 0.25 cm residual tumor tissue (complete cytoreduction);
• CC-2 ‒ 0.25–2.5 cm residual tumor tissue (incomplete cytoreduction with moderate residual tumor proportion);
• CC-3 ‒ > 2.5 cm residual tumor tissue (incomplete cytoreduction with high residual tumor proportion).

The Consensus^6^ was achieved on the pathologic classification of PMP, defined as the intraperitoneal accumulation of mucus due to mucinous neoplasia characterized by the redistribution phenomenon and classified:

1 Mucin without epithelial cells.
2 PMP with Low-grade. Low-grade mucinous peritoneal carcinoma or Dissemination Peritoneal Adenomatosis (DPAM).
3 PMP with High-grade. High-grade mucinous carcinoma peritonei or Peritoneal Mucinous Carcinomatosis (PMCA).
4 PMP with signet ring cells. High-grade mucinous carcinoma peritonei with signet ring cells OR Peritoneal Mucinous Carcinomatosis with Signet ring cells (PMCA-S).

Intraoperative adjuvant treatment can be applied through Peritoneal Hyperthermic Chemotherapy (HIPEC). The technique described by Spratt et al.^7^ Mitomycin, Oxaliplatin, or Cisplatin chemotherapy are currently used intraoperatively, which have been heated for 42 degrees.

## Objective

To evaluate the efficacy and safety in the application of intra-abdominal hyperthermic chemotherapy and cytoreductive surgery for patients with pseudomyxoma peritonei from the cecal appendix.

## Methods

The protocol of this study has been registered in PROSPERO (CRD42021252820). This systematic review will be prepared according to recommendations contained in PRISMA (Preferred Reporting Items for Systematic Reviews and Meta-Analyses).^8^

The eligibility criteria of the studies are:

1 Adult patient with PMP from cecal appendix;
2 Treatment – CRS and HIPEC;
3 Outcomes ‒ Mortality, disease-free survival, and adverse events of any cause, degree ≥ 3;^9^
4 Follow-up time up to 60-months;
5 Randomized controlled trials, comparative non-randomized studies and case series;
6 No period or language limit;
7 Full text available for access.

The search for evidence will be conducted on the following virtual scientific information databases, using the search strategies:

Medline/PubMed: ([Pseudomyxoma peritonei OR syndrome of pseudomyxoma peritoneal OR gelatinous ascites] AND [hyperthermic intraperitoneal chemotherapy]);

Central Cochrane: (Pseudomyxoma peritonei AND hyperthermic intraperitoneal chemotherapy).

The information obtained from the characteristics of the studies were: 'author's name and year of the study, study design, number of patients, population, methods of intervention and comparison, absolute number of outcomes, and follow-up.

The measurement used to express benefit and damage varied according to outcomes expressed by means of continuous variables (mean and standard deviation) or expressed by categorical variables (absolute number of events). In continuous measurement, the results are of difference in means and standard deviation, and in categorical measures, the results are of absolute risks, differences in risks, and number needed to treat or to produce damage, considering the number of patients. The confidence level used will be 95%. When in the presence of common outcomes among the included studies, the results will be expressed through meta-analysis.

### Bias assessment and quality of evidence

Case series studies or before and after will have their risk of bias analyzed according to the Joanna Briggs Institute Critical instrument.^10^ Cohort and case-control studies will be evaluated with the Robins *–* I instrument^11^ tool, while randomized clinical trials will have their risk of bias analyzed using the RoB 2 instrument.^12^

The results of comparative observational clinical trials will be aggregated and meta-analyzed using Revman 5.4^13^ software, while non-comparative studies will be meta-analyzed using the Comprehensive Metanalysis software.

Furthermore, the quality of evidence will be graded as high, moderate, low, or very low using the Grade instrument^14^ and considering the risk of bias, the presence of inconsistency, inaccuracy, or indirect evidence in the meta-analysis of the outcomes, and the presence of publication bias.

## Results

**Fig. 2:**
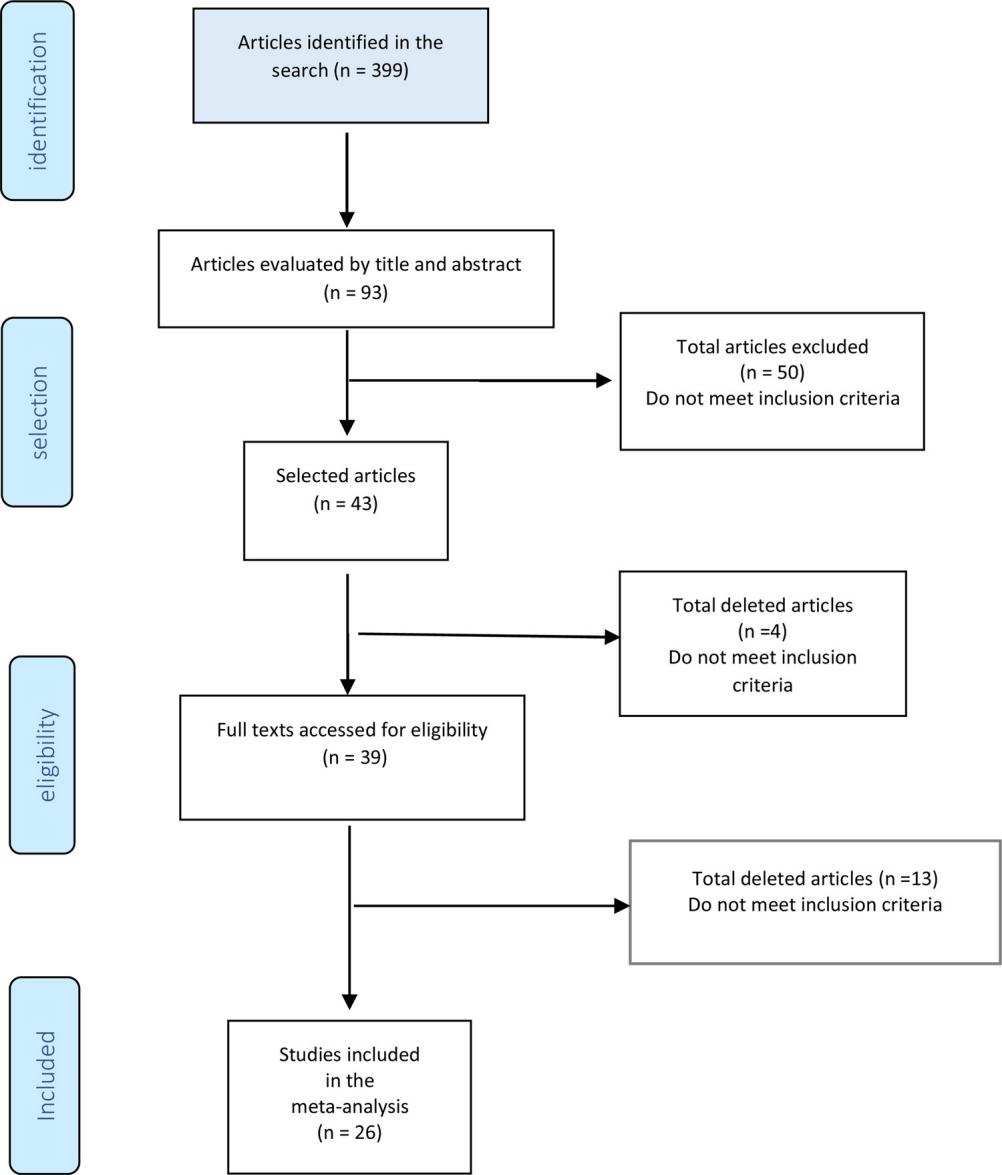
Flow diagram.

Fig. 10 shows the study diagram. As of January 2021, the search strategy identified 399 studies with titles and abstracts, and screening identified 94 potentially eligible citations. The full-test screening of 43 citations identified 26 studies^15^, ^16^, ^17^, ^18^, ^19^, ^20^, ^21^, ^22^, ^23^, ^24^, ^25^, ^26^, ^27^, ^28^, ^29^, ^30^, ^31^, ^32^, ^33^, ^34^, ^35^, ^36^, ^37^, ^38^, ^39^, ^40^ as potentially relevant publications, all studies were case series. The reasons for exclusion and the list of excluded studies are available in the references, ANNEXES (Fig. 2 and Table 1). The result was extracted in absolute numbers and meta-analyzed in absolute risk, without comparison.

**Table 1:** Excluded articles and reason for exclusion.

Study	Reason for exclusion
Austin 2015	Follow-up time 24-months
Auer 2020	Systematic review
Bratt 2017	Follow-up time 15-months
Bartoška 2020	Full article not found
Goslin 2012	Follow-up time 14-months
Hovath 2018	Follow-up time 18-months
Järvinen 2014	Did not apply HIPEC to all patients
Kusamura 2006	Phase II study
Kusamura 2019	Compares HIPEC infusion pressure
Kusamura 2014	Outcome evaluates learning curve
Leigh 2019	Outcome evaluates learning curve
Murphy 2007	Perioperative primary outcome
Mizumoto 2012	Follow-up time 30-days
Narasimhan 2019	Follow-up of 104 and 120-months
Narasimhan 2020	Follow-up time 18-months
Sugarbaker 2006	Intraoperative morbidity and mortality
Tabrizian 2014	Does not meet inclusion criteria
Van 2019	Outcome assesses prognostic factors
Van Leeuwen 2007	Follow-up time 24-months

The present study included population was a total of 3.274 patients with PMP from the cecal appendix, submitted to HIPEC and CCR treatment, followed for analysis of outcomes death, disease-free survival, and adverse effects in a mean follow-up of 36 and 60 months. Characteristics of the selected studies are described in Table 2, in annexes.

**Table 2:** Description of the included studies RCC associated with HIPEC in peritoneal pseudomyxoma originating from the cecal appendix.

Study	Design	Patient	Intervention	Comparison	Outcome	Follow-up
Alzahrani 2015	Case series (n = 675)	Patients undergoing CRS+HIPEC with peritoneal carcinomatosis of different origins	CRS+HIPEC (Source-dependent CT).	Index of carcinomatosis	Morbidity and mortality	60 months
				Grading of malignancy		
Azzam 2017	Case series (n = 38)	Patients with PMP undergoing CRS + HIPEC	CRS+HIPEC (Mitomycin, some CT before or after CRS)	Gender, PCI, SC, surgical time, histological grade, and blood loss.	Disease-free survival, mortality, and complications	Average of 54 months (1‒84)
Brandley 2006	Case series (n = 101)	Patients with PMP of origin in cecal appendix	CRS+HIPEC (mitomycin)	Prognosis in relation to histopathological classification	Mortality	36 and 60 months
Deraco 2006	Case series (n = 75)	Patients with PMP of origin in cecal appendix	CRS + HIPEC (mytomicin + cisplatinun)	Prognostic factors	Morbidity and mortality	Average of 37 months
Elias 2008	Case series (n = 105)	Patients with PMP of origin cecal appendix (88%) and another 12%	CRS+HIPEC (oxaliplatin or oxiplatin + irinotecan and 5 FU + leucovorin pre HIPEC)	PCI, Histopathologic and markers	Morbidity and mortality	Average of 48 months
Elias 2010	Case series (n = 301)	Patients with PMP in appendix (91%) and ovary 7%	CRS+HIPEC (mitomycin and oxaliplatin) and some cases EPIC (fluorouracil for 4 days) intraperitonandal)	Surgical classification, histology, sex, institution and HIPEC	Morbidity and mortality	Average of 88 months
Huang 2016	Case series (n = 250)	Patients with low-grade PMP submitted to CRS + HIPEC	CRS+HIPEC (mitomycin)	EPIC (CT post operation, 5-fluoracil, 2‒6 days)	Disease-free survival, mortality, and complications	60-months
Huang 2017	Case series (n = 185)	Patients with peritoneal adenocarcinoma of cecal appendix	CRS+HIPEC or CRS + HIPEC + EPIC (CT)	HIPEC + EPIC	Disease-free survival, mortality, and complications	60-months
Iversen 2013	Case series (n = 80)	Patients with peritoneal carcinomatosis (Colorectal, mesum and appendix origin) submitted to CRS + HIPEC	CRS + HIPEC (mitomycin or cisplatin)	Types of origin of carcinomatosis	Morbidity and mortality	Average of 26 months
Jimenez 2014	Case series (n = 202)	Patients with peritoneal carcinomatosis of appendix	CRS + HIPEC (does not inform chemotherapy used)	Histological type, PCI, lymph node involvement and surgery classification	Morbidity and mortality	60-months
Lansom 2016	Case series (n = 345)	Patients with pseudomyxoma from cecal appendix	CRS+HIPEC (Mitomycin, se PMCA) (oxaliplatin + folinic acid + 5FU[IV])	Surgical classification	Morbidity and mortality	60-months
Li 2020	Case series (n = 254)	Patients with pseudomyxoma from cecal appendix	CRS+HIPEC (cisplatin and mitomycin or cisplatin and docetaxel)	HIPEC, PCI, transfusion, and intra-operative blood loss	Morbidity and mortality	60-months
López-López 2017	Case series (n = 17)	Patients over 74 years old with PMP undergoing CRS + HIPEC	CRS+HIPEC (Mitomycin (by itself or in combination with Doxorubicin, paclitaxel and oxaliplatin))	Degree of complications, CRS efficacy	Disease-free survival, mortality, and complications	36-months
Lord 2015	Case series (n = 512)	Patients with PMP originating from perforation of mucinous tumor from cecal appendix	CRS+HIPEC (mitomycin)	Patients without recurrence. Patients with recurrence and reoperated. Patients with non-operated recurrence	Morbidity and mortality	60-months
Marcotte 2014	Case series (n = 58)	Patients with appendix carcinomatosis and PMP	CRS+HIPEC (oxaliplatin) + CT for PMCA (5-fluorouracil with irinotecan or oxaliplatin)	Histological types	Morbidity and mortality	Average of 33.7 months
				Results post-first intervention.		
Masckauchan 2019	Case series (n = 92)	Peritoneal appendix carcinomatosis	Peritonectomy + HIPEC (Oxiplatin)	Histological type	Morbidity and mortality	Average of 42 months
Munoz Zuluaga 2018	Case series (n = 151)	Patients with peritoneal carcinomatosis of high-grade from appendix origin	CRS + HIPEC (mitomycin)	Histological type (signet and non-signet) and abdominal lymph nodes	Morbidity and mortality	Average of 50 months
Nikiforchin 2020	Case series (n = 121)	Patients with low-grade appendix neoplasms	CRS + HIPEC (mitomycin)	Cellularity in low-grade PMP mucin	Mortality	120 months
Polanco 2016	Case series (n = 97)	Patients with mucinous neoplasms of high-grade cecal appendix and large volume of carcinomatosis	CRS+HIPEC (mitomycin + EPIC)	Volume of disease in high-grade PMP:	Morbidity and mortality	Average of 50.8 months
				High Volume Results (SPCI) ≥ 12 vs. Low Volume (SPCI) < 12		
Sinukumar 2019	Case series (n = 91)	Peritoneal pseudomyxoma	Peritonectomy + HIPEC (Mitomycin and/or CT (oxaliplatin and 5-FU-based)	Histological types of origin (appendix, ovary, colorectal, mesus)	Morbidity and mortality	36 months
Smeenk 2007	Case series (n = 103)	Patients with peritoneal pseudomyxoma with appendix (92%) and others (11%)	CRS + HIPEC (mitomycin), CT carcinoma (5 FU + leucovorin)	Prognostic factors	Disease-free survival, Morbidity, and mortality	Average of 51 months
Stewart 2006	Case series (n = 110)	Patients with cecal appendix carcinomatosis	CRS + HIPEC (mitomycin)	Prognostic factors	Morbidity and mortality	Average of 34.8 months
Sugarbaker 1999	Case series (n = 385)	Patient with peritoneal tumor dissemination of cecal appendix	CRS + HIPEC (mitomycin), systemic CT (5 FU + leucovorin)	CRS + HIPEC (mitomycin), EPIC (5 FU + leucovorin)	Morbidity and mortality	Average of 37 months
Vaira 2009	Case series (n = 53)	Patients with peritoneal pseudomyxoma	CRS+HIPEC ([mitomycin and cisplatinum] in cases of adeno-carcinomatosis, pre-surgical CT)	Surgical classification, histopathological type, and systemic CT.	Morbidity and mortality	60 months
Virzì 2012	Case series (n=26)	Patients with PMP	CRS + HIPEC (cisplatin + mitomycin)	Histological types	Morbidity and mortality	60 months
Youssef 2011	Case series (n = 456)	Patients with peritoneal pseudomyxoma from appendix cecal origin	CRS+HIPEC (mitomycin and some cases-5-fluorouracil for 4-days intraperitoneal)	Surgical classification	Morbidity and mortality	Average of 32 months

CRS, Cytoreductive Surgery; HIPEC, Intraperitoneal Chemotherapy; PCI, Peritoneal Carcinomatosis Index; CT, Chemotherapy; PMP, Peritoneal Pseudomyxoma; SC, Surgical Classification; EPIC, Early Postoperative Intraperitoneal Chemotherapy; PMCA, Peritoneal Mucinous Carcinomatosis; SPCI, Simplified Peritoneal Cancer.

NiKiforchin et al.,^32^ evaluated as prognostic factor cellularity in ascytic fluid in low-grade PMP: defined as acellular or cellular ascitic liquid, in the extraction of the results, both outcomes were added. Sugarbaker and Chang^37^ evaluated complete and incomplete cytoreductive surgery, the results used for meta-analysis were only from complete surgery. Munhoz-Zuluaga et al.,^31^ evaluated High-Grade Peritoneal Mucinous Carcinoma (HGMCP) and High-Grade Peritoneal Mucinous Carcinoma with Synet cells (HGMCP-S). During the study data extraction, both results were added to the outcomes in HGMCP and HGMCP-S. Polanco et al.,^33^ evaluated High-Volume (HV) disease as defined as SPCI C < 12, while SPCI > 12 was considered Low-Volume (LV) disease, and the results used were the sum of both for high-grade PMP outcomes. Huang Y et al.,^22^ evaluated patients with PMP without histopathological classification, submitted to HIPEC or HIPEC associated with Perioperative Chemotherapy (EPIC) (2‒6 days), data were collected only from patients submitted to HPIEC.

The judgments for the risk of bias of the 26 studies^15^, ^16^, ^17^, ^18^, ^19^, ^20^, ^21^, ^22^, ^23^, ^24^, ^25^, ^26^, ^27^, ^28^, ^29^, ^30^, ^31^, ^32^, ^33^, ^34^, ^35^, ^36^, ^37^, ^38^, ^39^, ^40^ were analyzed by the Joanna Briggs Institute Critical^10^ instrument: 80% presented low risk, 16% moderate risk, and 4% high risk. Results were summarised in a risk of bias graph (Table 3).

**Table 3:** Description of the biases of the included studies, for peritoneal pseudomyxoma of cecal appendix origin. Criteria of Joanna Briggs Institute Critical.

Study	Alzahnani	Azzam	Brandley	Deraco	Elias	Elias	Huang	Huang	Iversen	Jimenez	Lansom J	Li XB	Lopes	Lord	Marcotte E	Masckauchan	Munoz-Zuluaga	Nikiforchin	Poçaco PM	Sinukumar	Smeenk	Stewart	Sugarbaker	Vaira	Virzi	Youssef
Checklist	2015	2017	2006	2006	2008	2010	2016	2017	2013	2014	2016	2020	207	2015	2014	2019	2018	2020	2016	2019	2017	2006	1999	2009	2012	2011
Were there clear criteria for inclusion in the case series?	Y	Y	Y	Y	Y	Y	Y	Y	Y	Y	N	Y	Y	Y	Y	Y	Y	Y	Y	Y	Y	Y	Y	Y	Y	Y
Was the condition measured in a standard, reliable way for all participants induced in the case series?	Y	Y	Y	Y	Y	Y	Y	Y	Y	Y	U	Y	Y	Y	Y	Y	Y	Y	Y	Y	Y	Y	U	Y	Y	Y
Were valid methods used for identification of the condition for all participants included in the case series?	Y	Y	Y	Y	Y	Y	Y	Y	Y	Y	Y	Y	Y	Y	Y	Y	Y	Y	Y	Y	Y	Y	U	Y	Y	Y
Did the case series have consecutive inclusion of participants?	U	Y	U	U	Y	Y	Y	Y	U	U	U	U	U	N	Y	Y	U	Y	Y	U	Y	U	U	U	U	U
Did the case series have complete inclusion of participants?	Y	U	Y	Y	Y	Y	Y	Y	U	U	Y	U	U	N	Y	Y	U	N	Y	U	U	Y	U	U	Y	U
Was there clear reporting of the demographist of the participants in the study?	Y	Y	Y	Y	Y	Y	Y	Y	Y	Y	U	Y	Y	Y	Y	Y	Y	Y	Y	Y	U	Y	N	N	Y	Y
Was there clear reporting of clinical information of the participants?	Y	Y	Y	Y	Y	Y	Y	Y	Y	Y	Y	Y	Y	N	Y	Y	Y	Y	Y	Y	U	Y	N	N	Y	Y
Were the outcomes or follow up results of cases clearly reported?	Y	Y	Y	Y	Y	Y	Y	Y	Y	Y	Y	Y	Y	Y	Y	Y	Y	Y	Y	Y	Y	Y	Y	Y	Y	Y
Was there clear reporting of the presenting site(s)/clink(s) demographic information?	U	Y	Y	N	Y	Y	Y	Y	Y	Y	Y	Y	Y	N	N	Y	N	Y	N	Y	N	Y	N	N	Y	N
Was statistical analysis appropriate?	Y	Y	Y	Y	Y	Y	Y	Y	Y	Y	Y	Y	Y	Y	Y	S	Y	Y	Y	Y	Y	Y	Y	Y	Y	Y

Y, Yes; N, Not; U, Unclear.

## Meta-analysis

### Low-grade pseudomyxoma

Meta-analysis of eleven clinical trials^15^^,^^17^^,^^24^^,^^25^^,^^28^^,^^29^^,^^32^^,^^35^, ^36^, ^37^^,^^39^ including 1043 participants found that HIPEC and CRS.

**Fig. 3:**
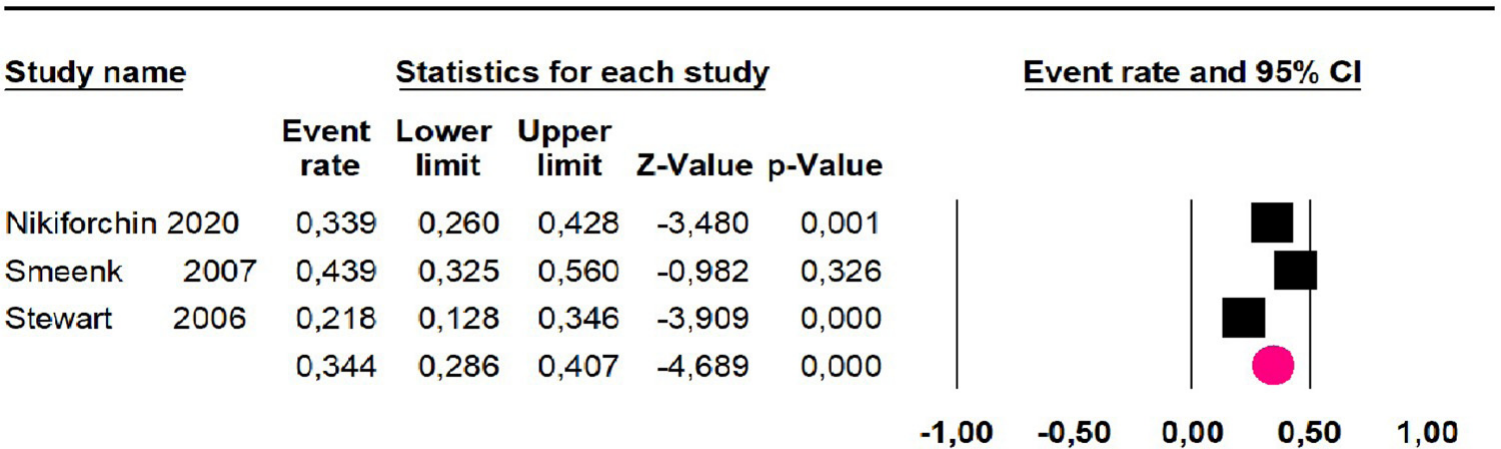
Comparison forest plot: low-grade pseudomyxoma, outcome: mortality at 36-months.

Mortality at 36-month was evaluated in three studies,^32^^,^^35^^,^^36^ including 242 participants. The risk of mortality was 34.4% (95% CI 28.6 and 40.7; I^2^ = 68.61%) (Fig. 3).

**Fig. 4:**
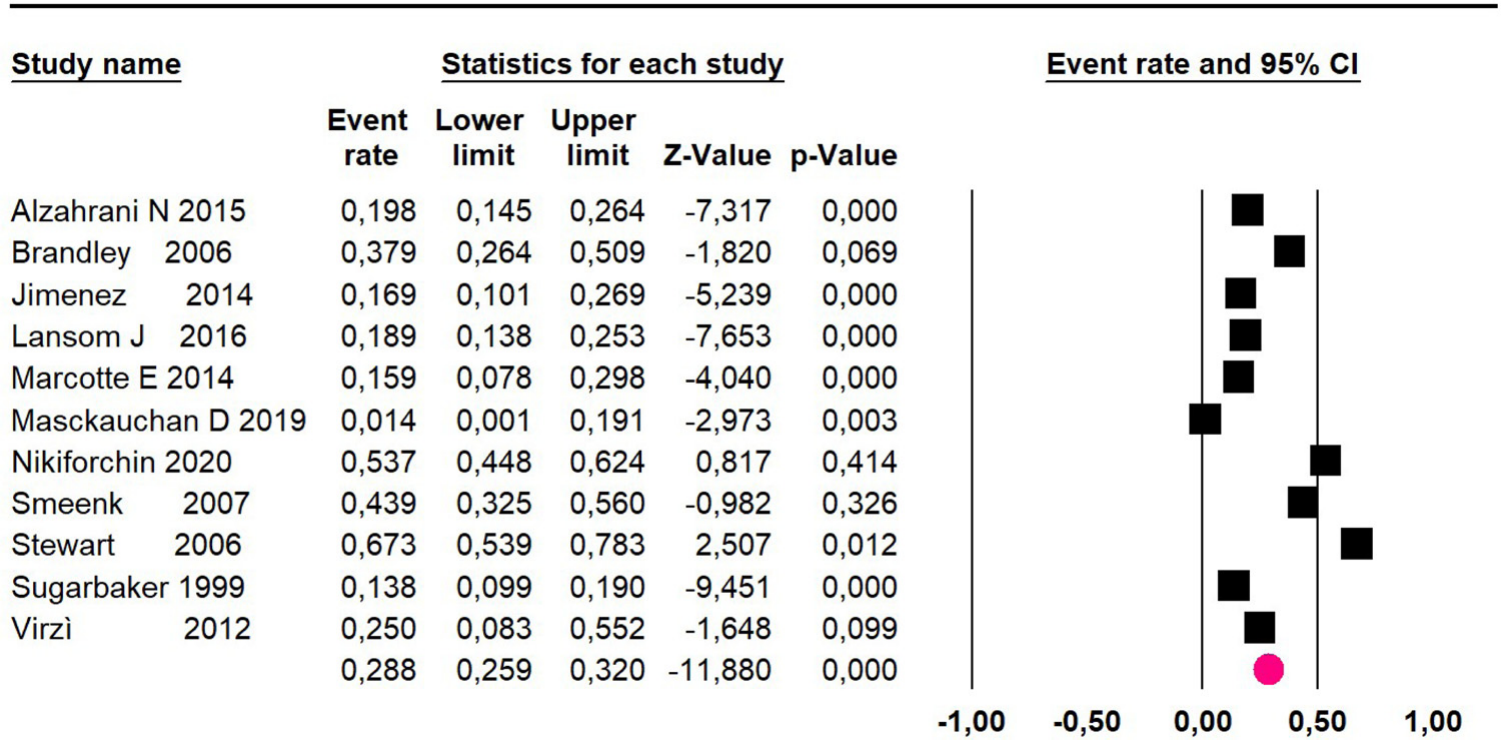
Comparison forest plot: low-grade pseudomyxoma, outcome: mortality at 60-months.

Mortality at 60-month: risk mortality was evaluated in eleven studies^15^^,^^17^^,^^24^^,^^25^^,^^29^^,^^30^^,^^32^^,^^35^, ^36^, ^37^^,^^39^ with 1043 patients. The risk was 28.8% (95% CI 25.9 to 32; I^2^ = 92.1%). Fig. 4.

**Fig. 5:**
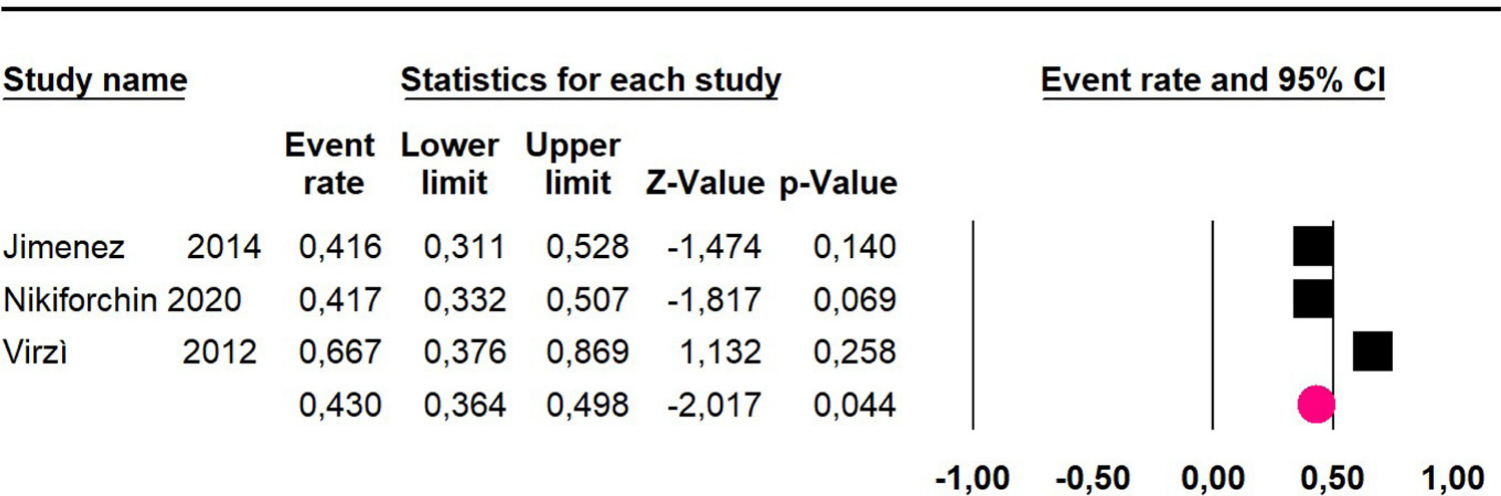
Comparison forest plot: low-grade pseudomyxoma, outcome: disease-free survival at 60-months.

Disease-free survival: Meta-analysis of three studies,^24^^,^^32^^,^^39^ assessing 209 participants, the follow-up 60-month risk was 43% (95% CI 36.4 and 49.8; I^2^ = 25.57%) (Fig. 5).

**Fig. 6:**
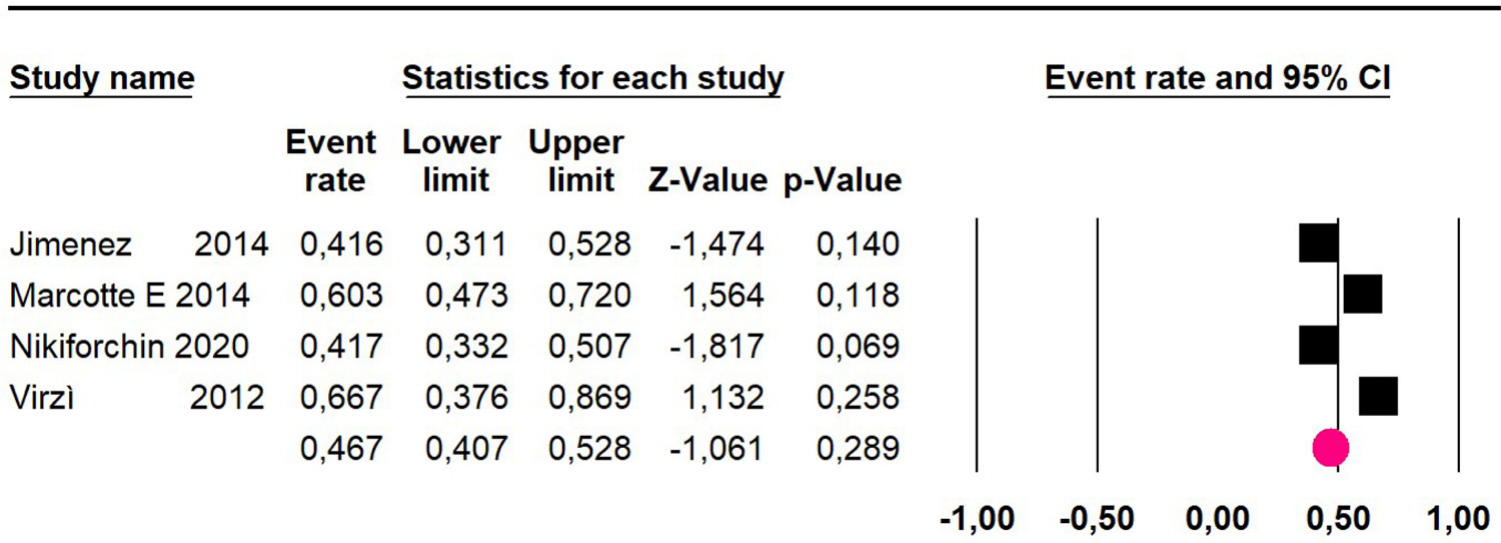
Comparison forest plot: low-grade pseudomyxoma, outcome: adverse events ≥3 at 60-months.

Adverse events greater than or equal to degree III: a meta-analysis of four studies^24^^,^^29^^,^^32^^,^^39^ with 267 patients, the 60-month risk was 46.7% (95% CI 40.7 to 52.8.3; I^2^ = 62.8%) (Fig. 6).

### High-grade pseudomyxoma

**Fig. 7:**
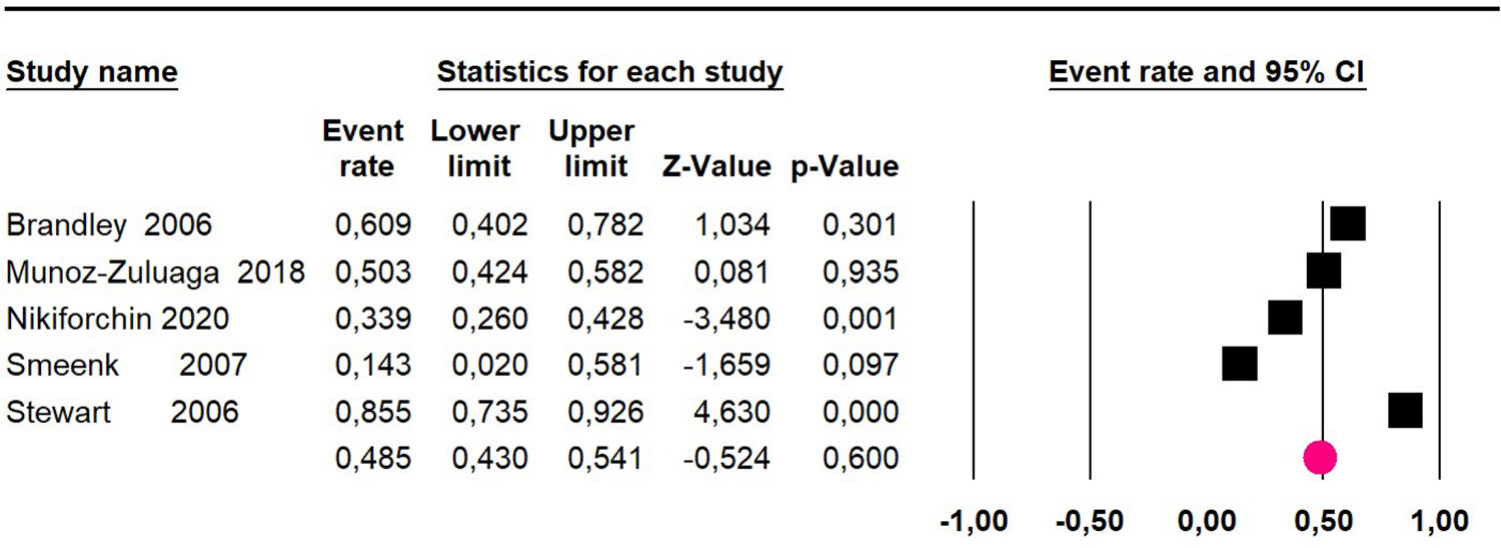
Comparison forest plot: high-grade pseudomyxoma, outcome: mortality at 36-months.
Mortality at 60-month: risk mortality was evaluated in nine studies^15^^,^^17^^,^^25^^,^^29^^,^^31^^,^^33^^,^^35^^,^^37^^,^^39^ including 772 patients, the risk was 55.9% (95% CI 52.1 to 59.6; I^2^ = 89.1%) (Fig. 8) between participants who have undergone HIPEC and CRS.

Meta-analysis of twelve studies,^15^^,^^17^^,^^24^^,^^25^^,^^29^^,^^30^^,^^32^^,^^33^^,^^35^^,^^36^^,^^37^^,^^39^ assessing 1073 participants, evaluated HIPEC and CRS for the outcome:

Mortality at 36-month was evaluated in five studies^17^^,^^31^^,^^32^^,^^35^^,^^36^ including 357 participants. The risk of mortality was 48.5% (95% CI 43% to 54.1%, I^2^ = 89.2%) (Fig. 7).

**Fig. 8:**
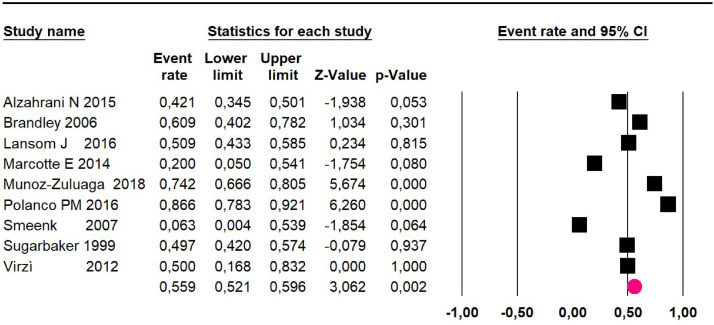
Comparison forest plot: high-grade pseudomyxoma, outcome: mortality at 60-months.
Disease-free survival: a meta-analysis of three studies,^24^^,^^31^^,^^33^ assessing 373 participants, the follow-up 36-month risk was 42.5% (95% CI 39.9 to 50.5; I^2^ = 94.13%) (Fig. 9) between participants who have undergone HIPEC and CRS.

**Fig. 9:**
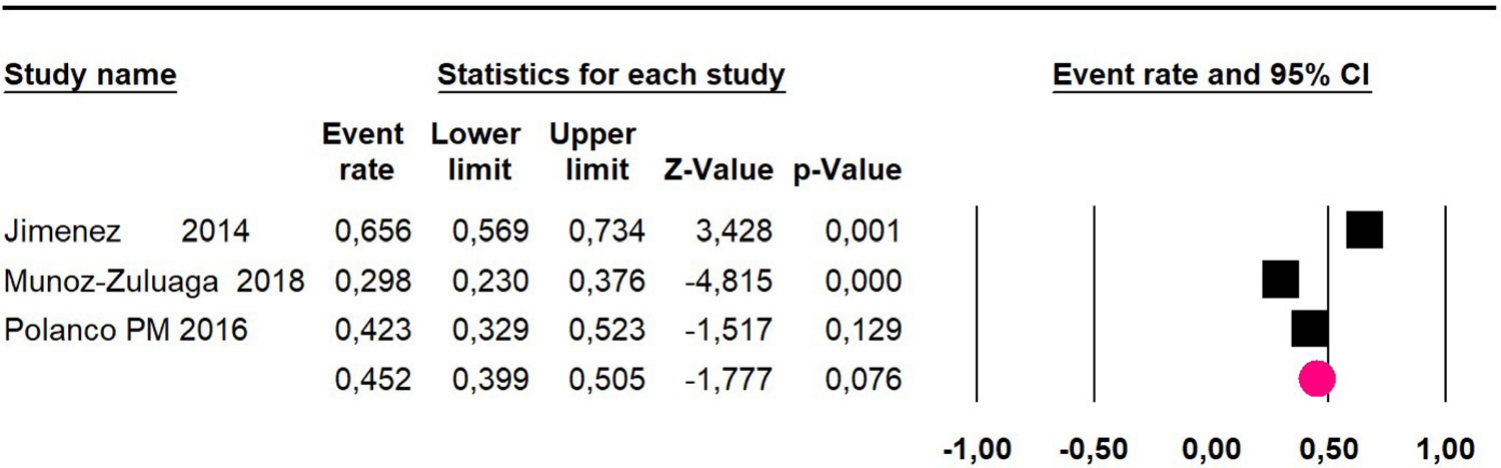
Comparison forest plot: high-grade pseudomyxoma, outcome: disease-free survival at 36-months.
The 60-month disease-free survival: a meta-analysis of three studies^31^^,^^33^^,^^39^ including 254 patients, reported risk 20.1% (95% CI 15.5 to 25.7; I^2^ = 70.84%) (Fig. 10) between participants who have undergone HIPEC and CRS.

**Fig. 10:**
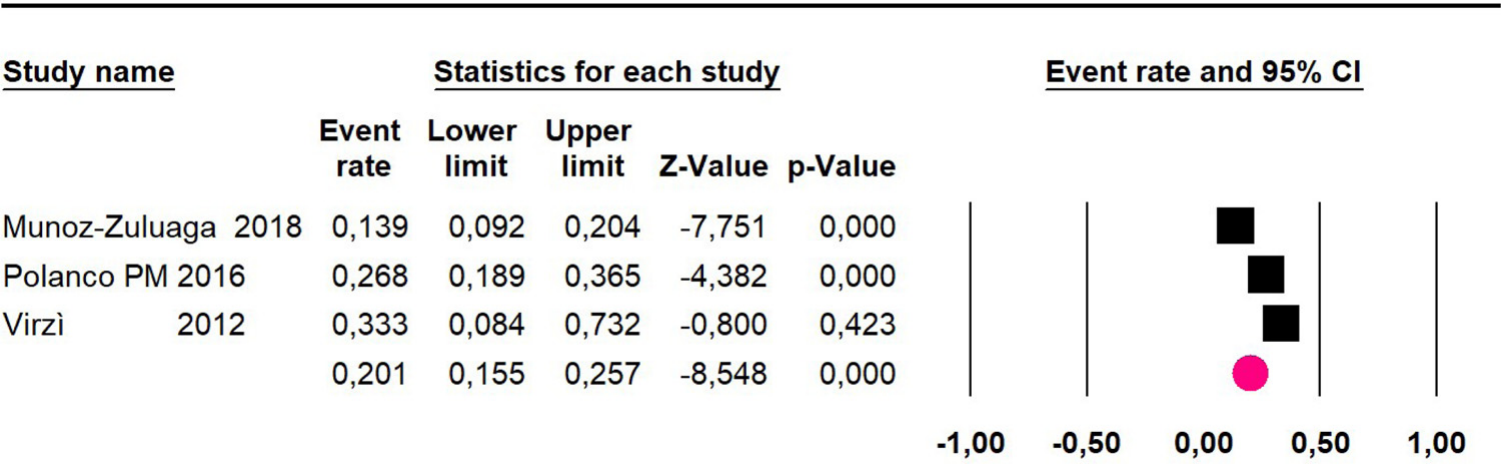
Comparison forest plot: high-grade pseudomyxoma, outcome: disease-free survival at 60-months.
Adverse events greater than or equal to grade III: a meta-analysis of four studies^24^^,^^29^^,^^33^^,^^38^ assessing 375 patients, reported 60-month risk of 30% (95% CI 25.2 to 35.3; I^2^ = 92.8%) (Fig. 11).

**Fig. 11:**
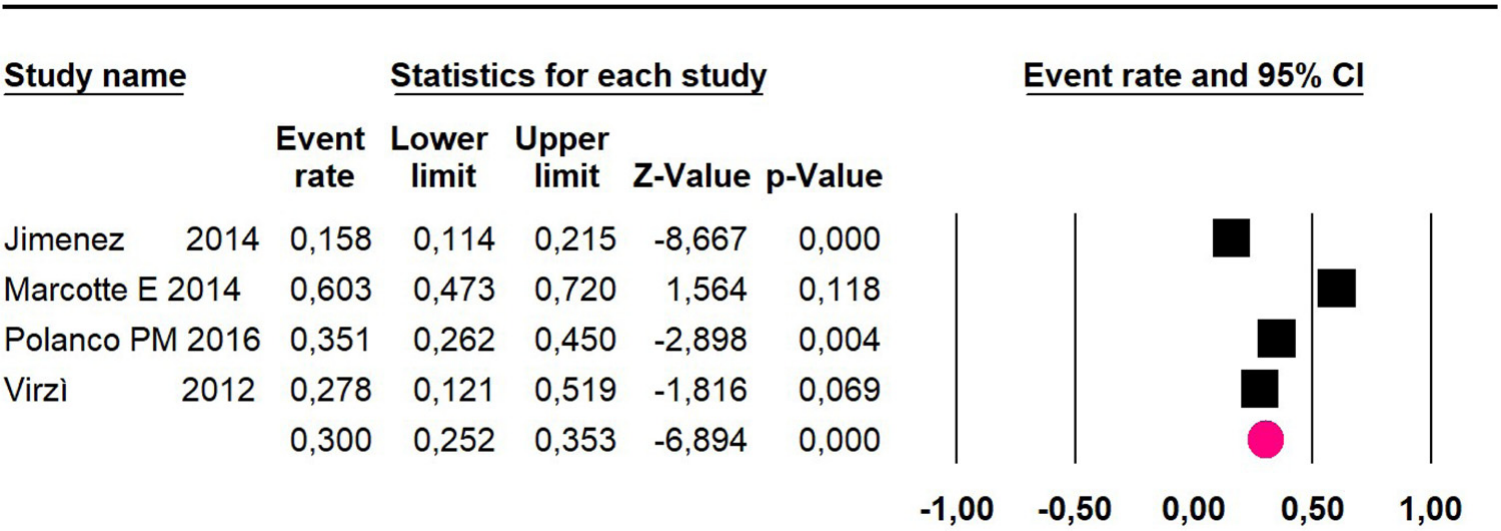
Comparison forest plot: low-grade pseudomyxoma, outcome: adverse events ≥3 at 60-months.

### Pseudomyxoma in general, without histopathological classification

**Fig. 12:**
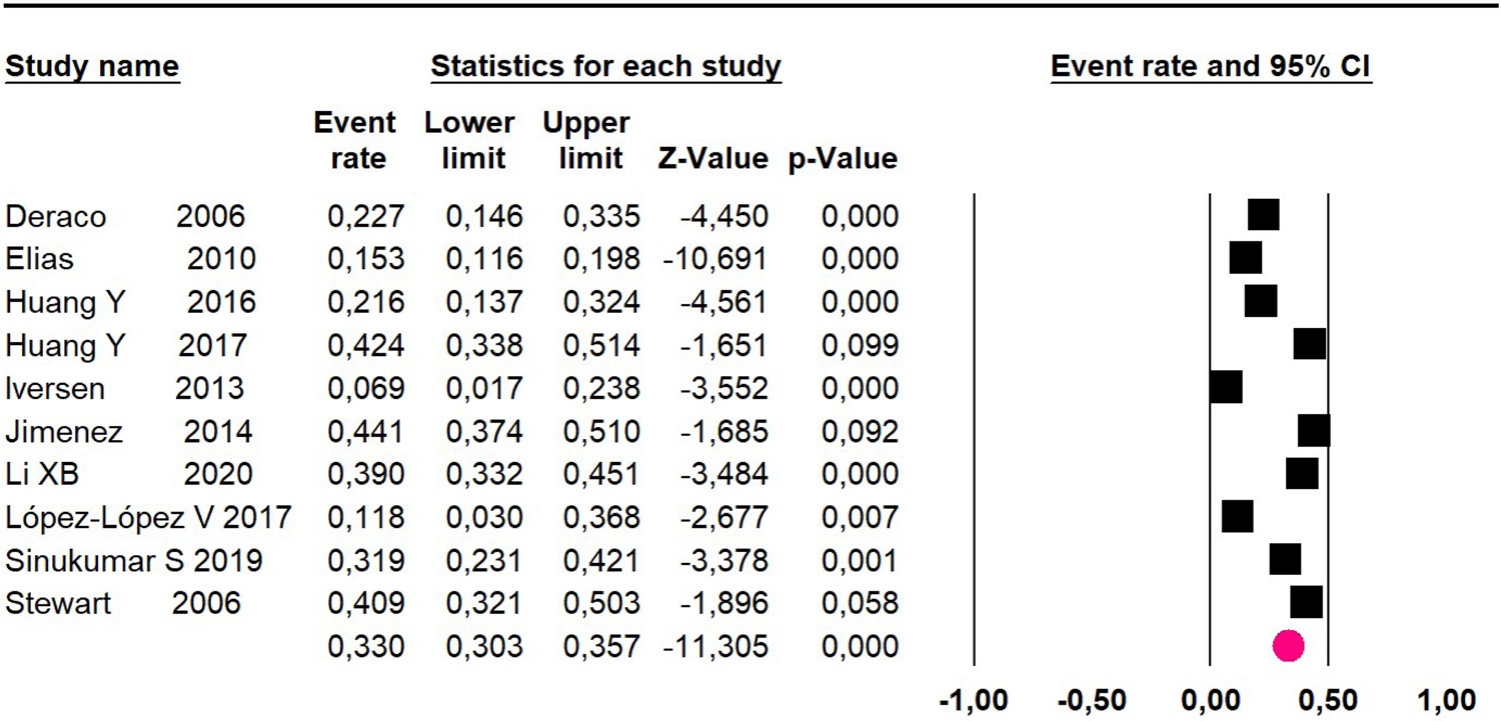
Comparison forest plot: without histopathological classification pseudomyxoma, outcome: mortality at 36-months.
Mortality at 60-month: risk mortality was evaluated in fourteen studies^13^^,^^16^^,^^17^, ^18^, ^19^, ^20^, ^21^, ^22^^,^^25^^,^^27^, ^28^, ^29^^,^^37^^,^^39^^,^^41^ [42] assessing 2209 patients, risk was 32.6% (95% CI 30.5 to 34.7; I^2^ = 94.45%) (Fig. 13) between participants who have undergone HIPEC and CRS.

Meta-analysis eighteen studies^16^^,^^18^, ^19^, ^20^, ^21^, ^22^, ^23^, ^24^^,^^26^, ^27^, ^28^, ^29^, ^30^^,^^34^^,^^36^^,^^38^, ^39^, ^40^ assessing 2594 participants evaluated HIPEC and CRS:

Mortality at 36-month was evaluated in ten studies^18^^,^^20^^,^^21^, ^22^, ^23^, ^24^^,^^26^^,^^27^^,^^34^^,^^36^ including 1271 patients. The risk was 33% (95% CI 30.3 to 35.7; I^2^ = 88.6%) (Fig. 12).

**Fig. 13:**
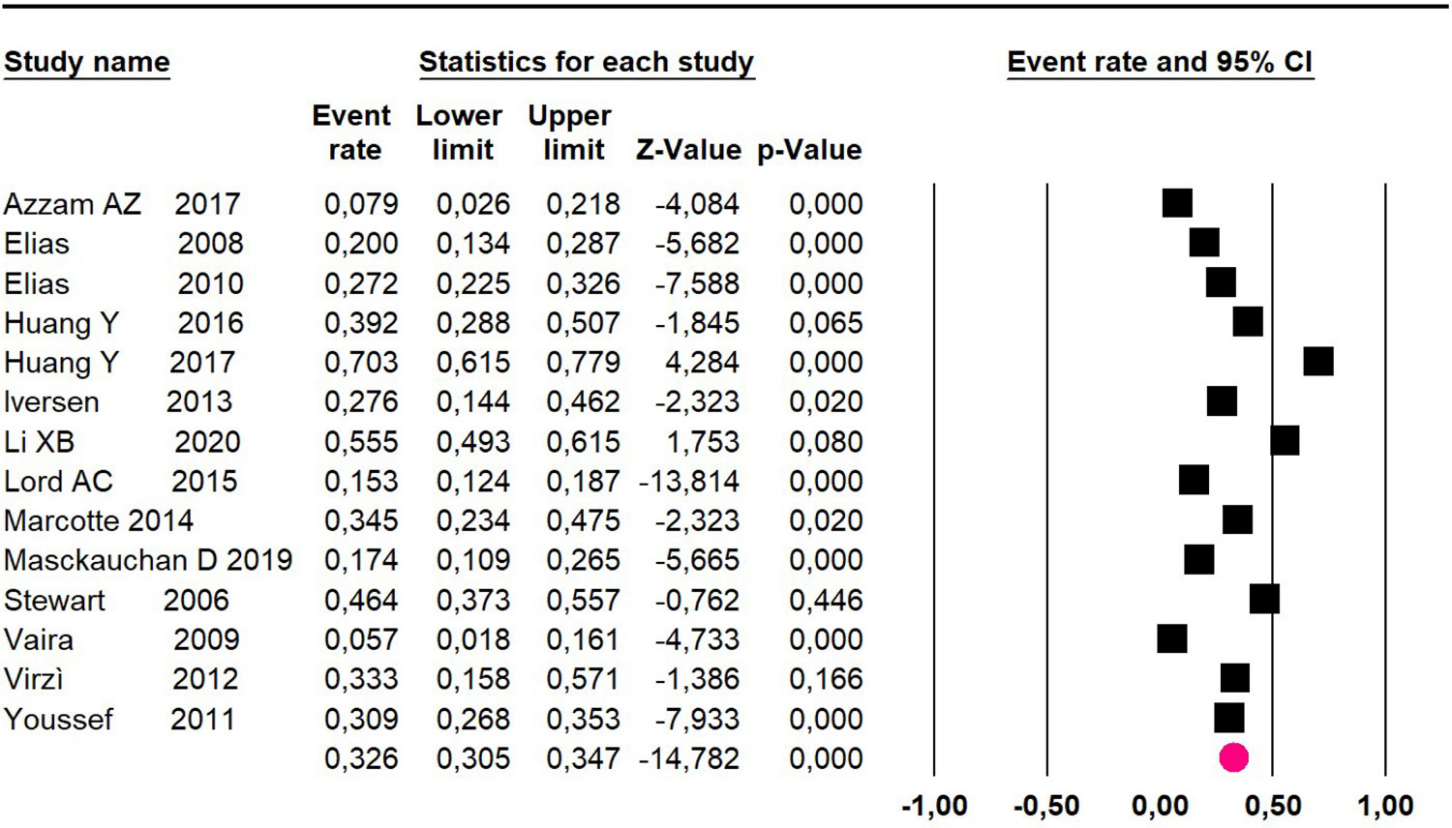
Comparison forest plot: without histopathological classification pseudomyxoma, outcome: mortality at 60-months.
Disease-free survival: meta-analysis of five studies^18^^,^^22^^,^^24^^,^^27^^,^^34^ including 503 participants, the follow-up 36-month risk was 50% (95% CI 45 to 55.1; I^2^ = 94.29%) (Fig. 14) between participants who have undergone HIPEC and CRS.

**Fig. 14:**
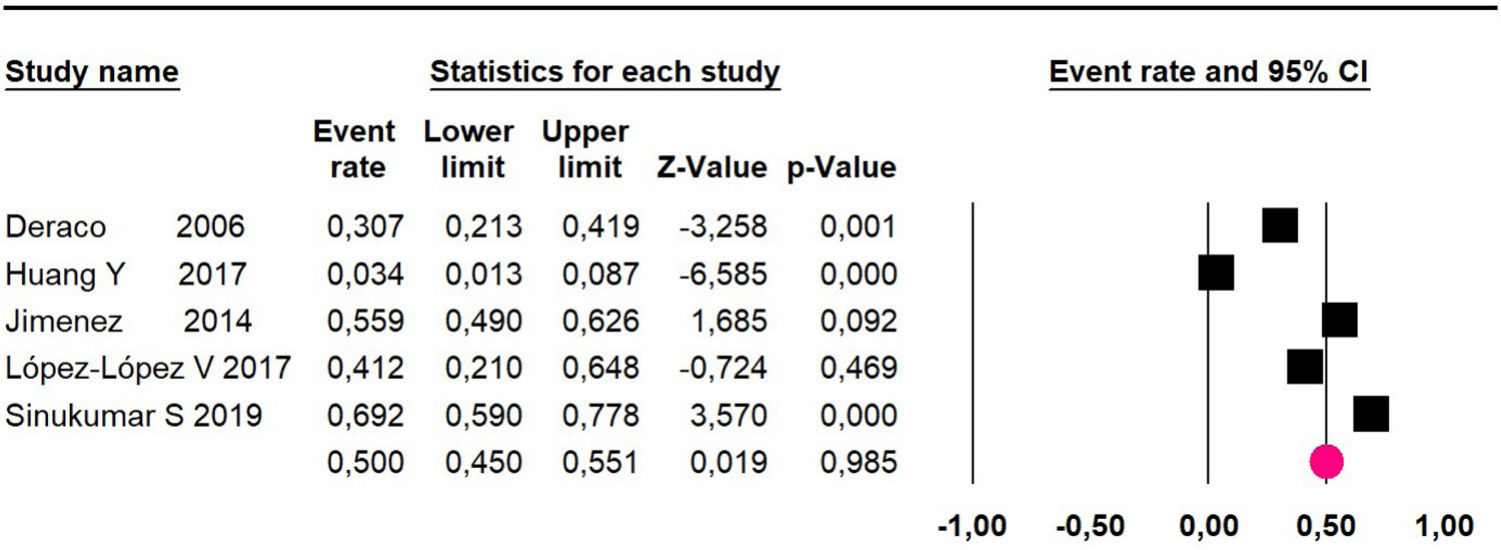
Comparison forest plot: without histopathological classification pseudomyxoma, outcome: disease-free survival at 36-months.
Disease-free survival: meta-analysis of other 9 studies^16^^,^^19^^,^^20^^,^^22^^,^^28^, ^29^, ^30^^,^^37^^,^^39^ including 1295 participants, reported risk of 61.8% (95% CI 58.8 to 64.7; I^2^ = 93.51%) (Fig. 15) at 60-month follow-up.

**Fig. 15:**
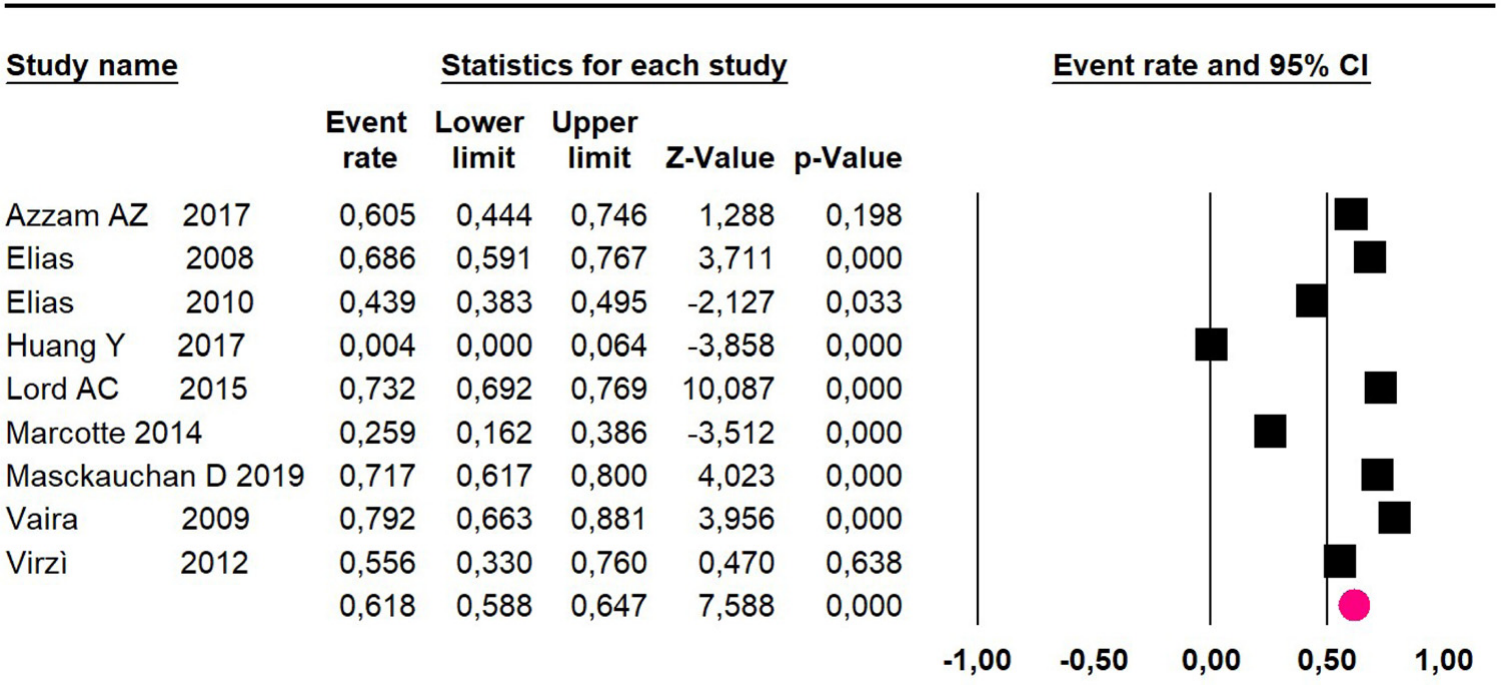
Comparison forest plot: without histopathological classification pseudomyxoma, outcome: disease-free survival at 60-months.
Adverse events greater than or equal to degree III: meta-analysis of 13^16^^,^^20^, ^21^, ^22^, ^23^, ^24^^,^^26^^,^^27^^,^^29^^,^^34^^,^^38^, ^39^, ^40^ studies reported adverse events to degree ≥ 3 for 1747 patients, the risk 60-month was 32.9% (95% CI 30.5 to 35.4; I^2^ = 93.58%) (Fig. 16).

**Fig. 16:**
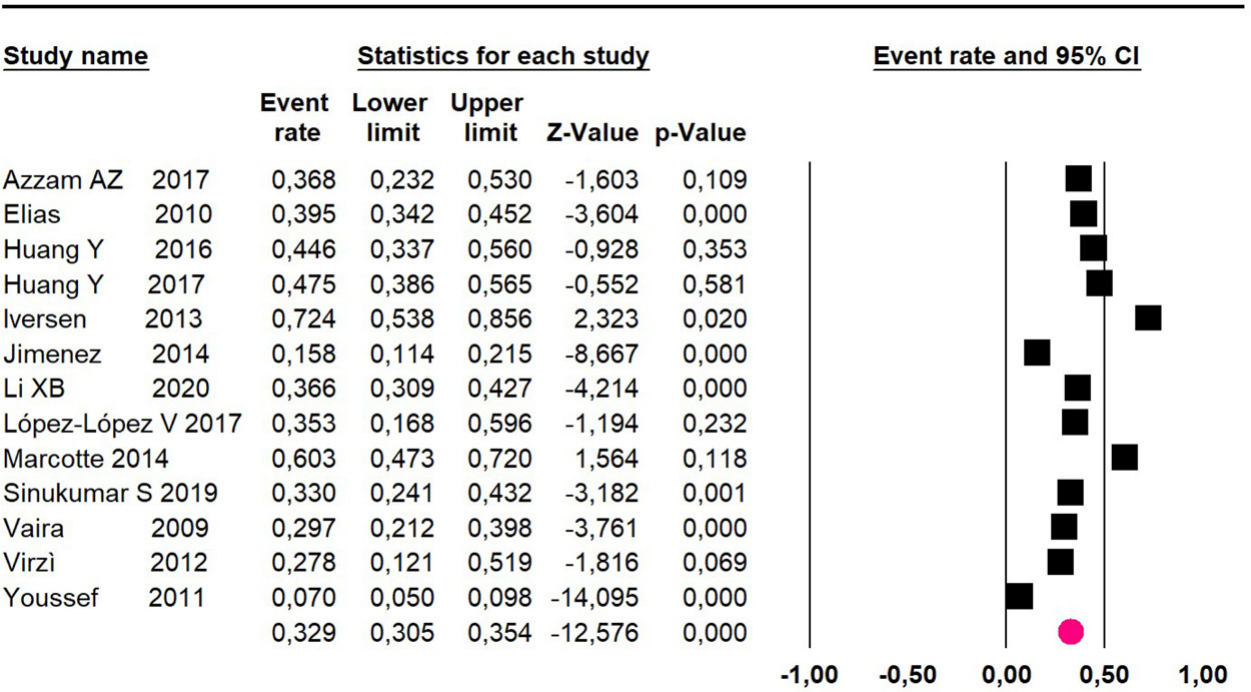
Comparison forest plot: without histopathological classification pseudomyxoma, outcome: adverse events ≥3 at 60-months.

### Quality of evidence

Quality of evidence was assessed using the GRADE instrument^14^ (Table 3) as very low quality for all outcomes, except for disease-free survival 60-month (low-grade PMP) outcome was low quality. Table 4

**Table 4:** Summary of results and analysis of evidence GRADE.^12^ Peritoneal pseudomyxoma cecal appendix origin.

N° of studies	Study design	Risk of bias	Inconsistency	Indirect ness	Imprecision	Other considerations	Risk of event	Quality	Importance
**Low-grade PMP. Mortality (follow-up: 36 months average)**									
3	Observational study	Not serious	Record^a^	Not serious	Not serious	None	34.4% (95% CI 28.6 to 40.7; I^2^ = 68.61%)	⨁◯◯◯ Very low	Important
**Low-grade PMP. Mortality (follow-up: 60 months average)**									
11	Observational study	Not serious	Very serious^b^	Not serious	Not serious	None	28.8% (95% CI 25.9 to 342; I^2^ = 92.1%)	⨁◯◯◯ Very low	Important
**Low-grade PMP. SLD (follow-up: 60 months. average)**									
4	Observational study	Not serious	Not serious	Not serious	Not serious	None	57% (95% CI 50.2 and 63.6; I^2^ = 25.57%)	⨁⨁◯◯ Low	Important
**Low-grade PMP. Adverse events (follow-up: 60 months average)**									
4	Observational study	Not serious	Very serious^c^	Not serious	Not serious	None	24.2% (95% CI 19.7 to 29.3; I^2^ = 94.7%)	⨁◯◯◯ Very low	Important
**Pmp high grade. Mortality (follow-up: 36 months average)**									
5	Observational study	Not serious	Serious^d^	Not serious	Not serious	None	48.5% (95% CI 43 to 54.1%; I^2^ = 89.2%)	⨁◯◯◯ Very low	Important
**Pmp high grade. Mortality (follow-up: mean 60 months)**									
8	Observational study	Not serious	Grave^e^	Not serious	Not serious	None	55% (95% CI 51.9 to 59.5; I^2^ = 89%)	⨁◯◯◯ Very low	Important
**Pmp high grade. SLD (follow-up: 36 months average)**									
3	Observational study	Not serious	Very serious^f^	Not serious	Not serious	None	45.6% (95% CI 25.7 to 67; I^2^ = 94.13%)	⨁◯◯◯ Very low	Important
**Pmp high grade. SLD (follow-up: 60 months average)**									
3	Observational study	Not serious	Very serious^g^	Not serious	Not serious	None	20.1% (95% CI 15.5 to 25.7; I^2^ = 70.84%)	⨁◯◯◯ Very low	Important
**Pmp high grade. Adverse events (follow-up: 60 months average)**									
4	Observational study	Not serious	Very serious^h^	Not serious	Not serious	None	33.1% (95% CI 16 to 56.3; I^2^ = 91.8%)	⨁◯◯◯ Very low	Important
**PMP without histopathological classification. Mortality (follow-up: 36 months average)**									
10	Observational study	Not serious	Very serious^i^	Not serious	Not serious	None	28.4% (95% CI 21 to 37.2; I^2^ = 88.91%)	⨁◯◯◯ Very low	Important
**PMP without histopathological classification. Mortality (follow-up: 60 months average)**									
14	Observational study	Not serious	Very serious^j^	Not serious	Not serious	None	29.2% (95% CI 21 to 39.2; I^2^ = 94.45%)	⨁◯◯◯ Very low	Important
**PMP without histopathological classification. SLD (follow-up: 36 months average)**									
5	Observational study	Not serious	Very serious^k^	Not serious	Grave^l^	None	35.1% (CI 95% 17 to 58.9; I^2^ = 94.29%)	⨁◯◯◯ Very low	Important
**PMP without histopathological classification. SLD (follow-up: 60 months average)**									
9	Observational study	Not serious	Very serious^m^	Not serious	Not serious	None	56% (95% CI 41.7 to 69.3; I^2^ = 93.51%)	⨁◯◯◯ Very low	Important
**PMP without histopathological classification. Adverse events (follow-up: 60 months average)**									
13	Observational study	Not serious	Very serious^n^	Not serious	Not serious	None	35% (95% CI 25.2 to 46.1; I^2^ = 93.58%)	⨁◯◯◯ Very low	Important

IC; Confidence Interval; I^2^ heterogeneity.

Explanations:

a Heterogeneity of 68.61%

b Heterogeneity 92.1%

c Heterogeneity 94.7%

d Heterogeneity 89.2%

e Heterogeneity 89%

f Heterogeneity 94.13%

g Heterogeneity 70.84%

h Heterogeneity 91.8%

i Heterogeneity 88.91%

j Heterogeneity 94.45%

k Heterogeneity 94.29%

I Confidence interval with wide amplitude; greater than two standard deviation

m Heterogeneity 93.51%

n Heterogeneidade 93.58%.

**Table 5:** Synthesis of evidence.

Outcomes	Low-grade PMP	High-grade PMP	PMP without histopathological classification
RM 36 months	34.4% (95% CI 28.6 to 40.7; I^2^ = 68.61%)	48.5% (95% CI 43 to 51.1%; I^2^ = 89.2%)	28.4% (95% CI 21 to 37.2; I^2^ = 88.91%)
RM 60 months	28.8% (95% CI 25.9 to 32; I^2^ = 92.1%)	55% (95% CI 52.1 to 59.6; I^2^ = 89.1%)	29.2% (95% CI 21 to 39.2; I^2^ = 94.45%)
SLD 36 months		45.6% (95% CI 25.7 to 67; I^2^ = 94.13%)	35.1% (95% CI 17 to 58.9; I^2^ = 94.29%)
SLD 60 months	57% (95% CI 50.2 to 63.6; I^2^ = 25.57%)	20.1% (95% CI 15.5 to 25.7; I^2^ = 70.84%)	56% (95% CI 41.7 to 69.3; I^2^ = 93.51%)
EAD 60 months	24.2% (95% CI 19.7 to 29.3; I^2^ = 94.7%)	33.1% (95% CI 16 to 56.3; I^2^ = 92.8%)	35% (95% CI 25.2 to 46.1; I^2^ = 93.58%)

RM, Mortality risk; EAD, Adverse Events.

### Summary of evidence (Table 5)

Low-grade PMP: mortality risk follow-up 36-month, 60-month, DFS 60-month, adverse events to degree ≥ 3 in 60-month follow-up risk was: 34.4% (95% CI 28.6 to 40.7; I^2^ = 68.61%); 28.8% (95% CI 25.9 to 32; I^2^ = 92.1%), 57% (95% CI 50.2 to 63.6; I^2^ = 25.57%) and 24.2% (95% CI 19.7 to 29.3; I^2^ = 94.7%).

High-grade PMP: mortality risk follow-up 36-month, 60-month, DFS 36-month, DFS 60-month, adverse events to degree ≥ 3 in 60-month follow-up risk was: 48.5% (95% CI 43% to 54.1%, I^2^ = 89.2%), 55.9% (95% CI 52.1 to 59.6; I^2^ = 89.1%), 45.6% (95% CI 25.7 to 67; I^2^ = 94.13%), 20.1% (95% CI 15.5 to 25.7; I^2^ = 70.84%); and 33.1% (95% CI 16 to 56.3; I^2^ = 92.8%).

PMP without histopathological classification: mortality risk follow-up 36-month, 60-month, DFS 36-month, DFS 60-month, adverse events to degree ≥ 3 in 60-month follow-up risk was: 28.4% (95% CI 21 to 37.2; I^2^ = 88.91%), 29.2% (95% CI 21 to 39.2; I^2^ = 94.45%), 35.1% (95% CI 17 to 58.9; I^2^ = 94.29%), 56% (95% CI 41.7 to 69.3; I^2^ = 93.51 and 35% (95% CI 25.2 to 46.1; I^2^ = 93.58%).

## Discussion

The absence of randomized and controlled studies results in the low incidence of the disease, 0.2 to 2 cases per 1.000.000 inhabitants per year.^41^ In the present systematic review, with meta-analysis, the authors found only a series of cases, the fact that compromises the quality of the evidence presented.

Historically the prognosis of peritoneal pseudomyxoma is associated with origin (ovary, mesus, uric, stomach, colon, and appendix), and Cytological grading of malignancy (adenomatous, carcinomatous, and intermediate) and peritoneal dispersion index.^5^

Currently, the treatment is performed through peritoneal cytoreduction with or without intrabdominal hyperthermic chemotherapy.

When the authors meta-analyze the low-grade PMP outcomes without histopathological classification, in 36-months, there was an observed improvement in survival for patients without histopathological classification, but in a 60-month outcome, there is a significant improvement in low-grade PMP patients; it can be justified by the slow progression of the disease in low-grade PMP in relation to high-grade, and it may increase the mortality in this group, reducing long-term survival.

When comparing DFS in the low-grade PMP groups and those without histopathological classification, in 60-months, the authors observed similar results, 57% and 56%, a fact that can be explained by the survival of patients with better surgical results, who are better likely to remain disease-free.

The studies evaluated individually present great differences between themselves, such as Masckauchan et al.,^30^ which reported a result of 0% in the mortality of patients with low-grade PMP in 60-months, while Smeenk et al.,^35^ presented mortality of 34% of the patients. This important variation between the results may be correlated with the sample number, the chemotherapeutic drug used, the clinical and demographic characteristics of patients, surgical classification, and experience of the surgical team in the execution of the procedure.

Currently, there are difficulties in commercializing mitomycin chemotherapeutic drugs, being the most used for the execution of HIPEC. Marcotte et al.^29^ and Masckauchan et al.^30^ analyzed the survival of patients with PMP submitted to CRS and HIPEC with oxaliplatin, chemotherapy of the same family as cisplatin and carboplatin, obtaining results similar to mitomycin, and therefore, it can be used during the HIPEC procedure.

## Conclusion

Peritoneal polymyxoma of the appendix is a rare disease with slow evolution and survival that depends on factors such as histological degree, peritoneal cytoreductive surgery and experience of the surgical team. Hyperthermic chemotherapy is recommended in selected cases with satisfactory results.

## Authors' contributions

Idevaldo F, Antonio S and Wanderley MB designed the study, performed the data collection and analysis, and critically reviewed the final version of the manuscript. João CR and Claudia C acquired some of the data. All authors read and approved the final version of the manuscript.

## Conflicts of interest

The authors declare no conflicts of interest.
